# The Association between Lifestyle Choices and Schizophrenia Symptoms

**DOI:** 10.3390/jcm10010165

**Published:** 2021-01-05

**Authors:** Sylwia Kalinowska, Beata Trześniowska-Drukała, Karolina Kłoda, Krzysztof Safranow, Błażej Misiak, Agnieszka Cyran, Jerzy Samochowiec

**Affiliations:** 1 Department of Psychiatry, Pomeranian Medical University, 26 Broniewskiego Street, 71-460 Szczecin, Poland; beata.trzesniowska@o2.pl (B.T.-D.); samoj@pum.edu.pl (J.S.); 2 Independent Laboratory of Family Physician Education, Pomeranian Medical University in Szczecin, 1 Rybacka Street, 70-204 Szczecin, Poland; wikarla@gazeta.pl; 3 Department of Biochemistry and Medical Chemistry, Pomeranian Medical University, 72 Powstancow Wlkp Street, 70-111 Szczecin, Poland; chrissaf@mp.pl; 4 Department of Psychiatry, Wroclaw Medical University, 10 Pasteura Street, 50-367 Wroclaw, Poland; mblazej@interia.eu (B.M.); agnieszka.cyran@umed.wroc.pl (A.C.)

**Keywords:** eating behavior, lifestyle, metabolic disorders, obesity, schizophrenia

## Abstract

Due to poor eating habits, insufficient physical activity, and nicotine use, schizophrenia patients are at increased risk of lifestyle diseases. Factors contributing to unhealthy behaviors include lower socioeconomic status and level of education as well as social isolation. Schizophrenia manifestations such as amotivation, apathy, and cognitive deficits can further hinder development of proper health habits. The aim of this study was to assess the possible association between lifestyle-related choices and schizophrenia symptoms severity. This observational study enrolled 106 patients with schizophrenia (42 Males/64 Females), 18–69 years (mean: 41.89 ± 9.7 years). Mean duration of schizophrenia was 14.61 ± 9.7 years. Multiple significant correlations were found between patients’ lifestyle and their biochemical laboratory parameters (lipid profile and fasting glucose). Most importantly, a significant link emerged between presented habits and schizophrenia symptom severity. There were also significant gender differences in the intake of sweets and sweet beverages. Quite unexpectedly, a behavioral shift towards more healthy lifestyle choices was observed after completion of questionnaires on lifestyle and health habits. There are clear benefits to systematic provision of educational interventions concerning physical activity and proper eating habits to schizophrenia patients. These simple preventive measures could significantly improve both mental and physical health outcomes in schizophrenia patient populations.

## 1. Introduction

Due to poor eating habits, insufficient physical activity, and nicotine use, schizophrenia patients are at increased risk for lifestyle diseases (such as hypertension, obesity, and type 2 diabetes) [1,2]. Factors contributing to unhealthy lifestyle include lower socioeconomic status and level of education as well as social isolation [3]. In addition, schizophrenia symptoms such as amotivation, apathy, and cognitive deficits, not to mention common side effects of antipsychotic drugs, can further hinder the development of proper health habits [4,5]. All these contribute to an increasing prevalence of obesity and its complications, leading to excess mortality observed in this patient population [4,6].

Individual dietary habits of schizophrenia patients are characterized by low consumption of fruit and vegetables with high intake of fat, sugar, and sodium [7]. While the effect of dietary intake on physical health status in the general population is widely known, the potential influence of diet on mental health in schizophrenia patients has been somewhat neglected, with existing research yielding inconsistent results. Nevertheless, most available evidence links unhealthy food choices to low socioeconomic status, cognitive deficits, and a preference for cheap, ready-made meals [8,9,10,11].

Cigarette smoking, which is more prevalent in schizophrenia patients (60–90%) than in the general population, is particularly detrimental to physical health in this population [12] and consistently proves to be the most difficult lifestyle habit to modify among this group. A long-term, 13-year study found that fatal diseases related to smoking were significantly more common in patients with schizophrenia relative to healthy controls [13]. The adverse effect of smoking on metabolic parameters confirms the aforementioned observation. Smokers have significantly higher levels of fasting glucose and triglycerides (TG) than nonsmokers [14]. On the other hand, nicotine use can reduce the negative effects of antipsychotics and/or improve cognitive performance in schizophrenia patients [15].

Lifestyle is known to affect not only the somatic health status, but also the severity of symptomatic manifestation in schizophrenia and vice versa—mental state impacts the everyday behavior [16,17]. This creates a vicious cycle that, if stopped, may provide certain health benefits for patients. Some studies found a negative association between physical activity with positive symptoms and general psychopathology [18] among schizophrenia patients, while negative symptoms were significantly associated with low self-reported physical activity [19]. There are also studies suggesting a significant association of metabolic syndrome and concentration of blood lipids with negative symptoms of schizophrenia [16,20]. The adoption of a healthy lifestyle is a process that involves motivation as well as physical, psychological, and material resources. Due to illness characteristics (i.e., impairments in cognition, perception, affect, and volition), schizophrenia patients have difficulties in each step of this process [21]. What is more, there is evidence that differences in sex-specific factors may play a role in this area and that individual nutritional education may reduce weight gain in patients with schizophrenia [22,23].

In view of the relative paucity of research on the subject, the aim of this study was to seek for the possible association between lifestyle-related choices (dietary and drinking habits, physical activity as well as smoking) and schizophrenia symptoms severity assessed with the Positive and Negative Syndrome Scale (PANSS).

## 2. Materials and Methods

This study was approved by the Bioethics Committee of the Pomeranian Medical University in Szczecin, Poland (approval number: KB-0012/72/11). All participants were informed about its purpose and terms. Written informed consent has been obtained from all participants, and the study was performed in accordance with the Declaration of Helsinki.

### 2.1. Study Design and Participants

This study had an observational character and was conducted without any intervention. A total of 106 patients with schizophrenia (42 Males/64 Females), 18–69 years (mean: 41.89 ± 9.7 years) were recruited in the years 2011–2016 across inpatient and outpatient psychiatric facilities and day care psychiatric wards at the Department of Psychiatry of the Pomeranian Medical University in Szczecin (Poland). After recruitment, only the outpatients took part in the study. Mean duration of schizophrenia was 14.61 ± 9.7 years. The International Statistical Classification of Diseases and Related Health Problems—version 10 (ICD-10) criteria were used to diagnose schizophrenia after evaluation of clinical history and medical records. Patients were assessed at two time points: at baseline (T1) and after follow-up observation lasting 8–10 weeks (T2).

Inclusion criteria comprised (1) a diagnosis of schizophrenia according to ICD-10 criteria; (2) age 18–69 years, and (3) ≥3 months treatment duration with a fixed dosage of antipsychotics. Exclusion criteria comprised dementia syndromes, a history of substance use disorders, severe cardiac disorders following a recent cardiovascular incident (minimum half a year after the incident) and/or congenital heart defects and/or arrhythmias (confirmed by history and electrocardigraphy—ECG), liver and/or kidney dysfunction (confirmed by history and laboratory tests), anemia, electrolyte imbalance, inflammatory diseases, oncologic diseases, prostate disease, pregnancy, thyroid disease treatment, and Cushing’s syndrome.

The majority of patients used atypical antipsychotic drugs (85.6%); however, doses of both classical and atypical antipsychotics have been subjected to conversion into chlorpromazine equivalents [24,25]. Doses of antipsychotics at baseline and after 8–10 weeks were not significantly different. Drop-out patients (*n* = 4) were not interested in further participation in the study; thus, they did not show up to the follow-up appointment. Table 1 presents clinical characteristics of the study sample at T1 (*n* = 106) and T2 (*n* = 102).

### 2.2. Psychometric Evaluation, Lifestyle Assessment, Anthropometric Measurements, Blood Pressure, and Biochemical Parameters

All patients underwent psychometric evaluation with the Positive and Negative Syndrome Scale (PANSS). This 30-item tool enables assessment of positive (P), negative (N), and general psychopathology (G) symptoms on a 7-point scale (1—absent, 2—minimal, 3—mild, 4—moderate, 5—moderately severe, 6—severe, and 7—extreme) [26].

Other data obtained via a self-designed patient survey concerned age, sex and illness duration, selected eating habits, sweet beverages consumption, nicotine use, and physical activity in the last 3 months.

Detailed questions regarded the following:

- weight of consumed sweets (in g/day),
volume of sweetened carbonated drinks (in mL/day),
amount of sugar used (in teaspoons/day),
volume of coffee and strong tea drunk per day (1 tea or coffee = 250 mL),
number of cigarettes smoked (no/day).

Physical activity of participants was evaluated on a three-point scale (adopted from World Health Organization—WHO), where

- 1 was low physical activity (sedentary lifestyle),
2 was moderate physical activity (physical activity performed to attend to routine daily duties)
3 was intense physical activity (physical activity undertaken at least 3× per week for ≥30 min).

Subsequent anthropometric measurements included weight, height, waist circumference, and body mass index (BMI). BMI was calculated using formula: body weight (kg)/height (m^2^). Blood pressure was measured twice on the right forearm using an appropriately selected cuff after 15 min of rest in a sitting position. The mean arterial pressure (MAP) was calculated with the formula MAP = 1/3 of systolic blood pressure (SBP) + 2/3 of diastolic blood pressure (DBP), using mean values of SBP and DBP from 2 measurements. Blood samples were collected to analyze selected biochemical parameters, including fasting blood glucose and lipid panel (total cholesterol (CHOL-T), low-density lipoproteins (LDL), high-density lipoproteins (HDL), and triglycerides (TG). Participants fasted for 12 h, and the blood samples were analyzed within 90 min. Glucose was determined by an enzymatic method with hexokinase on the Roche Cobas 6000 analyzer. The CE and CHOD/POD enzymatic-colorimetric methods were used to indicate CHOL-T. The HDL cholesterol fraction was determined by the PEG KYOWA method, MEDEX, and the LDL fraction by the enzymatic colorimetric method. The LPL enzyme-colorimetric method and peroxidase were used to evaluate TG concentration. All lipids fractions were assessed using a Roche COBAS 6000 analyzer. Follow-up appointments, attended by 102 patients (63 females and 39 males), were scheduled after approximately 8–10 weeks from baseline. Although the same author’s questionnaire was used at T1 and at T2, the participants at T2 were not asked about their behavior from the last 3 months, but from the baseline assessment point (last 8–10 weeks).

It should be emphasized that our study protocol did not include any lifestyle-changing interventions, and hence patients did not receive education on healthy lifestyle at baseline or during the observation period, nor were there any significant changes introduced to their psychiatric or somatic treatment. However, they could get acquainted with their laboratory test results and were free to consult a general practitioner or psychiatrist, if they expressed such desire.

### 2.3. Statistical Analysis

Statistica software (version 12, StatSoft Inc, Krakow, Poland) was used to perform all statistical analyses. Given the non-normal distribution of the majority of analyzed variables (*p* < 0.05, the Shapiro–Wilk test), we reached for the following nonparametric tests: the Wilcoxon matched pairs test to compare selected parameters at two time points, the Mann–Whitney U test and Pearson chi-square test to conduct comparisons between independent groups, the Spearman’s rank correlation coefficient to assess the relationship between continuous variables, and the two-sided Fisher’s exact test to analyze the relationships between dichotomous variables. The statistical power of the study with 100 participants was sufficient enough to detect significant correlation with 80% probability if the true effect size for association in the studied population corresponded to a correlation coefficient equal to ±0.28. Statistical significance was set at *p* < 0.05.

## 3. Results

### 3.1. General Observations

As mentioned earlier, although no lifestyle interventions were provided to the patients, they tended to voluntarily change their behavior after completing all the questionnaires. Given the disproportionate numbers of T2 and T1 participants, we could not perform statistical comparisons, but found a positive change in the number of cigarettes smoked per day, consumed coffee and sodas, and especially intake of sugar and sweets (15.2 ± 35.67 vs. 31.88 ± 56.20). Interestingly, and much in line with the observed habit changes, fasting blood glucose decreased (94.23 ± 20.85 vs. 99.16 ± 35.55) in T2 patients (Table 1). Incidentally, some of them completely gave up consumption of sugar and sweets (66 T1 vs. 51 T2 patients and 43 T1 vs. 25 T2 patients, respectively). Compared to baseline, there was a significant improvement in the PANSS assessment of all—positive, negative, and general—symptoms.

### 3.2. Correlations between Smoking, Eating Habits, Physical Activity and Biochemical Parameters

Having analyzed selected lifestyle-related factors in regard to blood lipids and fasting blood glucose, we found significant correlations between the number of smoked cigarettes and cholesterol levels at both T1 and T2: positive with CHOL-T and LDL, and negative with HDL (Table 2). We also observed positive correlations between the amount of consumed sweets and sweet beverages (sodas) and TG, and negative correlation with HDL. Those were, however, restricted to baseline assessments. Due to lifestyle changes and reduced consumption of sweets and sodas reported after the initial part of the study, there were no significant correlations with blood lipids at follow-up (T2). Similarly, consumed sugar correlated with CHOL-T and LDL only at T1, with no significant correlations at follow-up. In the case of consumed coffee, we observed significant positive correlations with CHOL-T and TG at T1, and with LDL at both T1 and T2. We also found significant correlations between physical activity and blood lipids—negative with TG at T1 and T2 and positive with HDL at T1. Observed correlations between physical activity and lipid levels are clearly beneficial and opposite to those previously described (Table 2).

### 3.3. Correlations between Smoking, Eating Habits, Physical Activity and PANSS Scores

The main aim of the study was to analyze the relationship between selected lifestyle habits and schizophrenia symptoms assessed with the PANSS. To that end, we found significant negative correlations between the number of cigarettes smoked daily and negative symptom severity at both T1 and T2. Moreover, at baseline, there emerged significant negative correlations between consumed sweets and the total, positive, and general symptom PANSS scores (*p* = 0.003, *p* = 0.012, and *p* = 0.0005, respectively). At follow-up, the only remaining significant negative correlation was observed in the case of general symptoms (*p* = 0.03). A similar observation was reported for soda consumption and general symptom severity at T2 (*p* = 0.045). What is more, we found significant negative correlations between the amount of consumed coffee and the total PANSS score as well as negative symptom severity at T1 (*p* = 0.034; *p* = 0.02).

The most interesting findings emerged in regard to physical activity. Initially, we found significant negative correlations between the level of physical activity and PANSS total, negative, and general symptoms scores (*p* = 0.014, *p* = 0.0006 and *p* = 0.019, respectively), which suggests that more intense physical activity was linked to less severe schizophrenia symptoms. This finding was even more pronounced at T2 (after re-assessment), where physical activity significantly correlated with every element of the PANSS—that is, total, positive, negative, and general symptom scores (*p* = 0.00002, *p* = 0.018, *p* < 0.00001, *p* = 0.00069, respectively) (Table 3).

### 3.4. Gender Differences

Based on literature reports that there are gender differences regarding lifestyle in schizophrenia patients, we performed a gender-specific lifestyle and PANSS scores comparison. Initially, men consumed significantly higher amounts of sweets and sweet beverages, compared with women. They also consumed more sugar and coffee, but these differences were not significant. There were also no significant differences in the severity of schizophrenia symptoms assessed with PANSS between men and women. However, after the follow-up, males cut down on sweets and sodas, but they still drank those beverages in significantly higher amounts than females. In addition, men achieved significantly higher PANSS scores in negative syndrome compared with women. There were differences, initially and after the follow-up, in levels of physical activity between males and females, but they did not reach the border of statistical significance (Table 4).

## 4. Discussion

The aim of this study was to explore the relationship between lifestyle (dietary habits and physical activity) and schizophrenia symptoms assessed with the PANSS. We found multiple significant correlations between patients’ lifestyle choices and their biochemical laboratory parameters (lipid panel and fasting glucose). Most importantly, we observed a significant relationship between reported habits and schizophrenia symptom severity. In addition, significant differences in the intake of sweets and sweet beverages emerged between male and female patients. Quite unexpectedly, a behavioral shift towards more healthy lifestyle choices was observed after completion of questionnaires on lifestyle and health habits.

In a study on 146 schizophrenia outpatients, Strassing et al. found that the dietary choices of male patients were not significantly different from those of healthy controls [9]. In contrast, relative to the control group, female patients reported significantly higher caloric intake in standard food components (proteins, carbohydrates, and lipids). Nevertheless, since it was not the aim of the authors to compare nutritional patterns of male and female patients with schizophrenia, their general conclusion was that regardless of gender, schizophrenia patients do not eat differently from the general population in terms of quality, but rather quantity [9]. On the other hand, in a later USA-based study on 88 schizophrenia patients, Hendersson et al. found that relative to the general population, schizophrenia patients reported lower total caloric intake and lower intake of fiber, folic acid, salt, and alcohol, but consumed significantly higher amounts of caffeine [10]. In a more recent study, Bly et al. observed that compared to patients with bipolar disorder, those with schizophrenia had significantly higher BMI, reported less physical activity, and smoked more cigarettes [27]. Quite remarkably, although quite similar in terms of research design and setting, those three studies yielded somewhat conflicting results. Possibly even more importantly, they seemed to have neglected the likely effect of lifestyle contributions on schizophrenia symptomatology. Therefore, our study was undertaken in an effort to fill this undisputed gap.

Available reports of the positive effect of wide and free access to fruit and vegetables on the dietary habits of schizophrenia patients suggest its rather temporary nature [28]. Calorie restriction alone, but even more so when combined with promotion of healthy eating habits, individual nutrition education, and motivation and encouragement to exercise appears to be effective in reducing weight gain and improving metabolic parameters in patients on antipsychotic medication [29,30,31]. We found that patients who consumed more sweets, sugar, sweetened beverages, and coffee had significantly worse lipid parameters in terms of TG, LDL, and HDL levels. Quite notably, the mere performance of a nutritional and health behavior interview resulted in reduced consumption of sweet beverages and improved metabolic parameters. Moreover, additional physical activity was significantly associated with the improvement of the lipid profile and blood glucose levels. Although taking up physical activity has a known positive effect on mental health, there is a relative paucity of research concerning its effect on schizophrenia patients, and in particular, reports are lacking on the impact of exercise on schizophrenia symptomatology and cognitive performance. Kimhy et al. conducted a randomized trial on the effect of an aerobic exercise program in 33 individuals with schizophrenia. After the completion of a 12-week exercise cycle, a significant improvement in neurocognitive performance was demonstrated in the patient population as compared with the control group [32]. An interesting report was also published by Malchow et al. in 2015. Namely, they observed a significant drop in the total PANSS score (0.037), as well as an improvement in both positive (*p* = 0.047 after 6 weeks; *p* = 0.035 after 3 months) and negative symptoms (*p* = 0.017) in response to undertaken physical activity in a sample of 43 schizophrenia patients [33]. This remains much in line with our findings concerning the observed significant relationship between physical activity and the severity of psychotic symptoms assessed with the PANSS, that is, reduced total PANSS score, as well as improved negative and general symptomatology.

Most schizophrenia patients smoke cigarettes. There are different hypotheses postulating the underlying mechanisms of this comorbidity [34,35]. Gage et al. found evidence consistent with a causal effect of smoking initiation on schizophrenia risk (OR 1.73, 95% CI 1.30–2.25, *p* < 0.001) [36]. Regarding the negative lifestyle habits, Dzien et al. showed that smokers had significantly higher fasting blood glucose (*p* < 0.01) and TG (*p* < 0.03) levels than nonsmokers [14]. Bobes et al. examined 1704 patients with schizophrenia and reported that smokers were more likely to consume alcohol daily (4.13 (3.07–5.54), *p* < 0.0001) and caffeine (3.39 (2.72–4.23), *p* < 0.0001) than nonsmoker patients with schizophrenia, and less likely to avoid daily consumption of salt (0.58 (0.43–0.78), *p* < 0.0001), saturated fat (0.71 (0.56–0.91), *p* = 0.006), high-fiber diet (0.67 (0.53–0.84), *p* = 0.001), or to follow a low-caloric diet (0.63 (0.48–0.81), *p* < 0.0001). Smokers also were less likely to do exercise habitually (0.62 (0.48–0.82, *p* = 0.001) [37]. The findings of An et al. suggest that schizophrenic patients who smoke have fewer psychotic symptoms, but contrary to expectation, smoking does not alter lipid profile levels [38].

In this study, a significant adverse effect of smoking on metabolic parameters was observed, especially on the lipid profile values. On the other hand, there is also evidence that smokers had significantly lower PANSS scores within the negative symptoms, which may be related to the relationship between smoking and the use of antipsychotics.

Despite Eugen Bleuler’s early description of abnormal eating behaviors in patients with schizophrenia, the effect of dietary choices on psychopathological manifestation remains poorly studied. In their 2019 study, Osuji and Onu analyzed eating habits in 206 patients with schizophrenia. They found that 13.2% of subjects exhibited abnormal feeding behaviors, which was associated with more severe positive symptoms [39]. Similarly, in a study on a sample of 87 schizophrenia patients, Adamowicz and Kucharska-Mazur indicated that less severe positive symptoms were associated with healthier eating behaviors [40].

Our study has some limitations. One of them is the use of authors’ questionnaires instead of standardized, validated food/eating habits questionnaires resulting from a lack of national nutritional guidelines in Poland. Another one is patient dropouts, but we have managed to keep this issue at a low level (3.8%). To minimize the bias, only one person interviewed and examined the patients, without providing them with lifestyle advice.

On the other hand, the strength of our study is the holistic assessment of dietary choices, anthropometric measurement, biochemical tests, and severity of disease symptoms instead of focusing only on one area of interest.

## 5. Conclusions

There are clear benefits to systematic provision of educational interventions concerning physical activity and proper eating habits to schizophrenia patients. These simple preventive measures could significantly improve both mental and physical health outcomes in schizophrenia patients.

## Figures and Tables

**Table 1:** Study participants clinical characteristics at baseline (*n* = 106) and after follow-up of 8–10 weeks (*n* = 102).

Measured Parameters	T1 (*n* = 106) Mean ± SD or %	T2 (*n* = 102) Mean ± SD or %
Height (cm)	169.9 ± 9.78	-
Body weight (kg)	84.3 ± 20.9	83.22 ± 19.78
BMI (kg/m^2^)	29.16 ± 8.86	29.16 ± 8.86
CIGARETTES (no/d)	8.01 ± 10.01	7.14 ± 9.20
COFFEE (mL/d)	1.11 ± 1.22	0.95 ± 1.00
TEA (mL/d)	1.04 ± 1.28	0.96 ± 1.23
SWEETS (g/d)	31.88 ± 56.20	15.2 ± 35.67
SODAS (mL/d)	69.33 ± 319.86	63.72 ± 324.20
SUGAR (tblsp/d)	1.57 ± 2.55	0.93 ± 1.49
Low physical activity (*N* of participants)	15/14.15	16/15.68
Moderate physical activity (*N* of participants)	71/66.98	64/62.74
Intense physical activity (*N* of participants)	20/18.86	22/21.56
CHOL-T (mg/dL)	193.54 ± 44.43	190.7± 40.35
TG (mg/dL)	159.27 ± 89	158.95 ± 90.39
LDL (mg/dL)	121.9 ± 37.18	120.17 ± 34.90
HDL (mg/dL)	48.15 ± 13.58	48.63 ± 13.34
Blood glucose (mg/dL)	99.16 ± 35.55	94.23 ± 20.85
SBP (mmHg)	121.46 ± 10.55	119.06 ± 9.27
DBP (mmHg)	74.67 ± 8.87	74.8 ± 7.92
PANSS (pt)	55.75 ± 21.19	44.45 ± 11.21
P (pt)	12.57 ± 6.78	9.23 ± 3.00
N (pt)	15.58 ± 6.36	12.71 ± 4.08
G (pt)	27.68 ± 10.64	22.60 ± 6.39

T1—baseline evaluation; T2—evaluation after 8–10 weeks; BMI—body mass index; Cigarettes—number of cigarettes smoked/d; COFFEE—amount of strong coffee/d; TEA—amount of strong tea/d; SWEETS- amount of sweets/d; SODAS—amount of consumed sweetened carbonated beverages/d; SUGAR—sugar intake/d; no—number; tblsp—tablespoon; *N*—number; CHOL-T—total cholesterol; TG—triglycerides; LDL—low-density lipoprotein; HDL—high-density lipoprotein; SBP—systolic blood pressure; DBP—diastolic blood pressure; PANSS—Positive and Negative Syndrome Scale; P—Positive Symptoms Scale; N—Negative Symptoms Scale; G—General Psychopathology Scale; pt—point.

**Table 2:** Correlations between smoking, eating habits, physical activity and biochemical parameters at T1 and T2.

Correlation	T1 (*n* = 106)		T2 (*n* = 102)	
	r	*p* *	r	*p* *
CIGARETTES and CHOL-T	0.19	**0.04**	0.23	**0.02**
CIGARETTES and TG	0.12	0.19	0.17	0.09
CIGARETTES and HDL	−0.21	**0.02**	−0.18	0.064
CIGARETTES and LDL	0.2	**0.03**	0.22	**0.03**
CIGARETTES and BLOOD GLUCOSE	−0.05	0.55	−0.16	0.11
SWEETS and CHOL-T	0.12	0.20	0.039	0.69
SWEETS and TG	0.27	**0.004**	0.076	0.44
SWEETS and HDL	−0.31	**0.0008**	−0.17	0.09
SWEETS and LDL	0.13	0.15	0.15	0.13
SUGAR and CHOL-T	0.23	**0.014**	0.17	0.088
SUGAR and TG	0.17	0.06	0.014	0.89
SUGAR and HDL	−0.09	0.35	0.099	0.32
SUGAR and LDL	0.20	**0.03**	0.085	0.40
SODAS and CHOL-T	−0.02	0.81	0.11	0.25
SODAS and TG	0.22	**0.01**	0.17	0.08
SODAS and HDL	−0.24	**0.01**	−0.19	0.05
SODAS and LDL	0.01	0.87	0.15	0.13
COFFEE and CHOL-T	0.21	**0.02**	0.19	0.055
COFFEE and TG	0.22	**0.02**	0.12	0.22
COFFEE and HDL	−0.15	0.11	−0.13	0.19
COFFEE and LDL	0.21	**0.02**	0.22	**0.028**
COFFEE and BLOOD GLUCOSE	0.09	0.34	−0.12	0.24
PHYSICAL ACTIVITY and CHOL-T	−0.16	0.09	−0.089	0.37
PHYSICAL ACTIVITY and TG	−0.25	**0.009**	−0.305	**0.002**
PHYSICAL ACTIVITY and HDL	0.21	**0.03**	0.17	0.09
PHYSICAL ACTIVITY and LDL	−0.18	0.05	−0.051	0.608
PHYSICAL ACTIVITY and BLOOD GLUCOSE	−0.14	0.14	−0.14	0.17

T1—baseline evaluation; T2—evaluation after 8–10 weeks; CIGARETTES—the number of cigarettes; COFFEE—amount of strong coffee; SWEETS- amount of sweets; SODAS—amount of consumed sweetened carbonated beverages; SUGAR—sugar intake; CHOL-T—total cholesterol; TG—triglycerides; LDL—low-density lipoprotein; HDL—high-density lipoprotein; *p* * calculated with Spearman’s rank correlation test; significant correlations are marked in bold characters.

**Table 3:** Correlations between smoking, eating habits, physical activity and PANSS score at T1 and T2.

Correlation	T1 (*n* = 106)		T2 (*n* = 102)	
	r	*p* *	r	*p* *
CIGARETTES and PANSS	−0.114	0.24	−0.11	0.25
CIGARETTES and P	0.010	0.91	0.018	0.86
CIGARETTES and N	−0.23	**0.018**	−0.20	**0.046**
CIGARETTES and G	−0.047	0.63	−0.11	0.26
SWEETS and PANSS	−0.28	**0.003**	−0.16	0.1
SWEETS and P	−0.24	**0.012**	−0.085	0.39
SWEETS and N	−0.15	0.12	−0.0501	0.62
SWEETS and G	−0.33	**0.0005**	−0.21	**0.03**
SODAS and PANSS	0.033	0.73	−0.12	0.24
SODAS and P	0.016	0.87	−0.12	0.24
SODAS and N	0.069	0.48	−0.0059	0.95
SODAS and G	0.046	0.64	−0.20	**0.045**
SUGAR and PANSS	−0.090	0.35	0.027	0.78
SUGAR and P	−0.031	0.75	0.12	0.21
SUGAR and N	−0.035	0.71	−0.043	0.66
SUGAR and G	−0.142	0.14	−0.0039	0.97
COFFEE and PANSS	−0.20	**0.034**	−0.08	0.38
COFFEE and P	−0.05	0.59	0.07	0.46
COFFEE and N	−0.22	**0.02**	−0.1	0.3
COFFEE and G	−0.21	0.02	−0.13	0.16
PHYSICAL ACTIVITY and PANSS	−0.24	**0.014**	−0.408	**0.00002**
PHYSICAL ACTIVITY and P	−0.052	0.59	−0.23	**0.018**
PHYSICAL ACTIVITY and N	−0.33	**0.0006**	−0.43	**0.000005**
PHYSICAL ACTIVITY and G	−0.22	**0.019**	−0.33	**0.00069**

T1—baseline evaluation; T2—evaluation after 8–10 weeks; CIGARETTES—the number of cigarettes; COFFEE—amount of strong coffee; SWEETS—amount of sweets; SODAS—amount of consumed sweetened carbonated beverages; SUGAR—sugar intake; PANSS—Positive and Negative Syndrome Scale; P—Positive Symptoms Scale; N—Negative Symptoms Scale; G—General Psychopathology Scale; *p* * calculated with Spearman’s rank correlation test; significant correlations are marked in bold characters.

**Table 4:** Gender-specific comparison of selected lifestyle choices and PANSS scores at T1 and T2.

			T1 (*n* = 106)			T2 (*n* = 102)		
	Parameter		F (*n* = 64)	M (*n* = 42)	*p*-Value	F (*n* = 63)	M (*n* = 39)	*p*-Value
			Mean ± SD or *N* (%)	Mean ± SD or *N* (%)		Mean ± SD or *N* (%)	Mean ± SD or *N* (%)	
HABITS	CIGARETTES (no/d)		8.01 ± 10.03	8.02 ± 10.11	0.94	6.90 ± 8.76	7.53 ± 9.98	0.89
	SWEETS (g/d)		23.25 ± 46.26	44.88 ± 67.18	**0.03**	10.87 ± 28.23	22.30 ± 44.70	0.1
	SODAS (mL/d)		6.25 ± 24.39	165.47 ± 494.40	**0.0009**	6.98 ± 27.39	155.38 ± 513.98	**0.01**
	SUGAR (tblsp/d)		1.48 ± 2.82	1.71 ± 2.08	0.25	0.76 ± 1.02	1.21 ± 2.01	0.59
	COFFEE (mL/d)		1.01 ± 1.17	1.26 ± 9.98	0.35	0.84 ± 0.93	1.12 ± 1.10	0.20
	* PHYSICAL ACTIVITY	LOW	6 (9.4)	9 (21.4)	0.086 *	6 (9.5)	10 (25.6)	0.09 *
		MODERATE	44 (68.8)	27 (64.3)		43 (68.3)	21 (53.8)	
		INTENSE	14 (21.9)	6 (14.3)		14 (22.2)	8 (20.5)	
PANSS	TOTAL SCORE (pt)		54.42 ± 20.05	57.73 ± 22.92	0.52	42.44 ± 8.70	47.69 ± 13.89	0.06
	P (pt)		12.31 ± 6.34	12.97 ± 7.46	0.88	8.93 ± 2.85	9.71 ± 3.21	0.24
	N (pt)		14.68 ± 5.75	16.95 ± 7.06	0.06	11.71 ± 2.73	14.33 ± 5.26	**0.01**
	G (pt)		27.45 ± 10.30	28.04 ± 1.24	0.94	21.67 ± 4.58	24.12 ± 8.39	0.32

T1—baseline evaluation; T2—evaluation after 8–10 weeks; CIGARETTES—number of cigarettes smoked in units/d; COFFEE—amount of strong coffee/d; SWEETS—amount of sweets/d; SODAS—amount of consumed sweetened carbonated beverages/d; SUGAR—sugar intake/d; PANSS—Positive and Negative Syndrome Scale; P—Positive Symptoms Scale; N—Negative Symptoms Scale; G—General Psychopathology Scale; SD—standard deviation; *p*-value of the Mann–Whitney U test; * *N*—number of participants (percentage of study sample) and *p*-value for Pearson chi-square test; significant correlations were marked in bold characters.

## Data Availability

Data sharing not applicable.

## References

[1] Ratliff J.C., Palmese L.B., Reutenauer E.L., Liskov E., Grilo C.M., Tek C. (2012). The effect of dietary and physical activity pattern on metabolic profile in individuals with schizophrenia: A cross-sectional study. Compr. Psychiatry.

[2] Vancampfort D., Knapen J., Probst M., van Winkel R., Deckx S., Maurissen K., Peuskens J., De Hert M. (2010). Considering a frame of reference for physical activity research related to the cardiometabolic risk profile in schizophrenia. Psychiatry Res..

[3] Heald A., Pendlebury J., Anderson S., Narayan V., Guy M., Gibson M., Haddad P., Livingston M. (2017). Lifestyle factors and the metabolic syndrome in Schizophrenia: A cross-sectional study. Ann. Gen. Psychiatry.

[4] Allison D.B., Newcomer J.W., Dunn A.L., Blumenthal J.A., Fabricatore A.N., Daumit G.L., Cope M.B., Riley W.T., Vreeland B., Hibbeln J.R. (2009). Obesity among those with mental disorders: A national institute of mental health meeting report. Am. J. Prev. Med..

[5] De Hert M., Detraux J., van Winkel R., Yu W., Correll C.U. (2011). Metabolic and cardiovascular adverse effects associated with antipsychotic drugs. Nat. Rev. Endocrinol..

[6] Capasso R.M., Lineberry T.W., Bostwick J.M., Decker P.A., St Sauver J. (2008). Mortality in schizophrenia and schizoaffective disorder: An Olmsted County, Minnesota cohort: 1950-2005. Schizophr. Res..

[7] Teasdale S.B., Ward P.B., Samaras K., Firth J., Stubbs B., Tripodi E., Burrows T.L. (2019). Dietary intake of people with severe mental illness: Systematic review and meta-analysis. Br. J. Psychiatry.

[8] Strassing M., Brar J.S., Ganguli R. (2003). Body mass index and quality of life in community-dwelling patients with schizophrenia. Schizophr Res..

[9] Strassing M., Brar J.S., Ganguli R. (2003). Nutritional assessment of patients with schizophrenia: A preliminary study. Schizophr. Bull..

[10] Henderson D.C., Borba C.P., Daley T.B., Boxill R., Nguyen D.D., Culhane M.A., Louie P., Cather C., Evins A.E., Freudenreich O. (2006). Dietary intake profile of patients with schizophrenia. Ann. Clin. Psychiatry.

[11] Wendołowicz A., Stefańska E., Jankowska D., Waszkiewicz N., Ostrowska L. (2020). Intake of selected nutraceuticals and the clinical condition of patients with mental disorders. Arch. Psychiatry Psychother..

[12] de Leon J., Diaz F.J. (2005). A meta-analysis of worldwide studies demonstrates an association between schizophrenia and tobacco smoking behaviors. Schizophr. Res..

[13] Rüther T., Bobes J., De Hert M., Svensson T.H., Mann K., Batra A., Gorwood P., Möller H.J. (2014). European Psychiatric Association. EPA guidance on tobacco dependence and strategies for smoking cessation in people with mental illness. Eur. Psychiatry.

[14] Dzien A., Dzien-Bischinger C., Hoppichler F., Lechleitner M. (2004). The metabolic syndrome as a link between smoking and cardiovascular disease. Diabetes Obes. Metab..

[15] Kumari V., Postma P. (2005). Nicotine use in schizophrenia: The self medication hypotheses. Neurosci. Biobehav. Rev..

[16] Chen S.F., Hu T.M., Lan T.H., Chiu H.J., Sheen L.Y., Loh E.W. (2014). Severity of psychosis syndrome and change of metabolic abnormality in chronic schizophrenia patients: Severe negative syndrome may be related to a distinct lipid pathophysiology. Eur. Psychiatry.

[17] Jakobsen A.S., Speyer H., Nørgaard H.C.B., Karlsen M., Hjorthøj C., Krogh J., Mors O., Nordentoft M., Toft U. (2018). Dietary patterns and physical activity in people with schizophrenia and increased waist circumference. Schizophr. Res..

[18] Wichniak A., Skowerska A., Chojnacka-Wójtowicz J., Tafliński T., Wierzbicka A., Jernajczyk W., Jarema M. (2011). Actigraphic monitoring of activity and rest in schizophrenic patients treated with olanzapine or risperidone. J. Psychiatr. Res..

[19] Nyboe L., Moeller M.K., Vestergaard C.H., Lund H., Videbech P. (2016). Physical activity and anomalous bodily experiences in patients with first-episode schizophrenia. Nord. J. Psychiatry.

[20] Kalinowska S., Trześniowska-Drukała B., Safranow K., Pełka-Wysiecka J., Kłoda K., Misiak B., Samochowiec J. (2019). Association between thyroid function and metabolic syndrome in male and female schizophrenia patients. Psychiatry Res..

[21] Hasnain M., Vieweg R.V.W. (2011). Do we truly appreciate how difficult it is for patients with schizophrenia to adapt a healthy lifestyle?. Acta Psychiatr. Scand..

[22] Chen S., Broqueres-You D., Yang G., Wang Z., Li Y., Yang F., Tan Y. (2016). Male sex may be associated with higher metabolic risk in first-episode schizophrenia patients: A preliminary study. Asian J. Psychiatry.

[23] Evan S., Newton R., Higgins S. (2005). Nutritional intervention to prevent weight gain in patients commenced on olanzapine: A randomized controlled trial. Aust. N. Z. J. Psychiatry.

[24] Andreasen N.C., Pressler M., Nopoulos P., Miller D., Ho B.C. (2010). Antipsychotic dose equivalents and dose-years: A standardized method for comparing exposure to different drugs. Biol. Psychiatry.

[25] Danivas V., Venkatasubramanian G. (2013). Current perspectives on chlorpromazine equivalents: Comparing apples and oranges!. Indian J. Psychiatry.

[26] Kay S.R., Fiszbein A., Opler L.A. (1987). The positive and negative syndrome scale (PANSS) for schizophrenia. Schizophr. Bull..

[27] Bly M.J., Taylor S.F., Dalack G., Pop-Busui R., Burghardt K.J., Evans S.J., McInnis M.I., Grove T.B., Brook R.D., Zöllner S.K. (2014). Metabolic syndrome in bipolar disorder and schizophrenia: Dietary and lifestyle factors compared to the general population. Bipolar Disord..

[28] McCreadie R.G., Kelly C., Connolly M., Williams S., Baxter G., Lean M., Paterson J.R. (2005). Dietary improvement in people with schizophrenia: Randomised controlled trial. Br. J. Psychiatry.

[29] Aquila R., Emanuel M. (2000). Interventions for weight gain in adults treated with novel Antipsychotics. Prim. Care Companion J. Clin. Psychiatry.

[30] Menza M., Vreeland B., Minsky S., Gara M., Radler D.R., Sakowitz M. (2004). Managing atypical antipsychotic-associated weight gain: 12-month data on a multimodal weight control program. J. Clin. Psychiatry.

[31] Wu M.K., Wang C.K., Bai Y.M., Huang C.Y., Lee S.D. (2007). Outcomes of obese, clozapine-treated inpatients with schizophrenia placed on a six-month diet and physical activity program. Psychiatr. Serv..

[32] Kimhy D., Vakhrusheva J., Bartels M.N., Armstrong H.F., Ballon J.S., Khan S., Chang R.W., Hansen M.C., Ayanruoh L., Smith E.E. (2014). Aerobic fitness and body mass index in individuals with schizophrenia: Implications for neurocognition and daily functioning. Psychiatry Res..

[33] Malchow B., Keller K., Hasan A., Dörfler S., Schneider-Axmann T., Hillmer-Vogel U., Honer W.G., Schulze T.G., Niklas A., Wobrock T. (2015). Effects of endurance training combined with cognitive remediation on everyday functioning, symptoms, and cognition in multiepisode Schizophrenia patients. Schizophr. Bull..

[34] Chen J., Bacanu S.A., Yu H., Zhao Z., Jia P., Kendler K.S., Kranzler H.R., Gelernter J., Farrer L., Minica C. (2016). Cotinine meta-analysis group, and FTND meta-analysis group (2016) genetic relationship between Schizophrenia and Nicotine dependence. Sci. Rep..

[35] Hu Y., Fang Z., Yang Y., Rohlsen-Neal D., Cheng F., Wang J. (2018). Analyzing the genes related to nicotine addiction or schizophrenia via a pathway and network based approach. Sci. Rep..

[36] Gage S.H., Jones H.J., Taylor A.E., Burgess S., Zammit S., Munafò M.R. (2017). Investigating causality in associations between smoking initiation and schizophrenia using mendelian randomization. Sci. Rep..

[37] Bobes J., Arango C., Garcia-Garcia M., Rejas J. (2010). Healthy lifestyle habits and 10-year cardiovascular risk in schizophrenia spectrum disorders: An analysis of the impact of smoking tobacco in the CLAMORS schizophrenia cohort. Schizophr. Res..

[38] An H.-M., Tan Y.-L., Tan S.-P., Shi J., Wang Z.-R., Yang F.-D., Huang X.-F., Soars J.C., Kosten T.R., Zhang X.-Y. (2016). Smoking and serum lipid profiles in Schizophrenia. Neurosci. Bull..

[39] Osuji P., Onu J. (2019). Feeding behaviors among incident cases of schizophrenia in a psychiatric hospital: Association with dimensions of psychopathology and social suport. Clin. Nutr. ESPEN.

[40] Adamowicz K., Kucharska-Mazur J. (2020). Dietary behaviors and metabolic syndrome in schizophrenia patients. J. Clin. Med..

